# Distinct roles of Fto and Mettl3 in controlling development of the cerebral cortex through transcriptional and translational regulations

**DOI:** 10.1038/s41419-021-03992-2

**Published:** 2021-07-14

**Authors:** Kunzhao Du, Zhen Zhang, Zhiwei Zeng, Jinling Tang, Trevor Lee, Tao Sun

**Affiliations:** 1 Center for Precision Medicine, School of Medicine and School of Biomedical Sciences, Huaqiao University, Xiamen, Fujian China; 2 School of Life Sciences and Biotechnology, Shanghai Jiao Tong University, Shanghai, China; 3 Department of Cell and Developmental Biology, Cornell University Weill Medical College, New York, NY USA

**Keywords:** Developmental neurogenesis, Transcriptomics, Neural progenitors

## Abstract

Proper development of the mammalian cerebral cortex relies on precise gene expression regulation, which is controlled by genetic, epigenetic, and epitranscriptomic factors. Here we generate RNA demethylase *Fto* and methyltransferase *Mettl3* cortical-specific conditional knockout mice, and detect severe brain defects caused by *Mettl3* deletion but not *Fto* knockout. Transcriptomic profiles using RNA sequencing indicate that knockout of *Mettl3* causes a more dramatic alteration on gene transcription than that of *Fto*. Interestingly, we conduct ribosome profiling sequencing, and find that knockout of *Mettl3* leads to a more severe disruption of translational regulation of mRNAs than deletion of *Fto* and results in altered translation of crucial genes in cortical radial glial cells and intermediate progenitors. Moreover, Mettl3 deletion causes elevated translation of a significant number of mRNAs, in particular major components in m^6^A methylation. Our findings indicate distinct functions of Mettl3 and Fto in brain development, and uncover a profound role of Mettl3 in regulating translation of major mRNAs that control proper cortical development.

## Introduction

The mammalian cerebral cortex plays a central role in controlling a range of behaviors such as perception, movement, cognition, memory, and emotion [1–4]. The cortex consists of diverse cell types and forms a well-organized inside-out laminar architecture, which is achieved through tight gene expression regulations during embryonic and postnatal development [5–7]. Neural progenitors reside in the ventricular zone (VZ) and subventricular zone (SVZ) proliferate and give rise to projection neurons, which migrate to the cortical plate (CP) and form six layers structure [7–12]. Altered production of neural progenitors and newborn neurons is associated with brain malformations and dysfunctions [13]. Numerous studies have demonstrated crucial roles of genetic and epigenetic factors in regulating proper cortical development [14–18]. Moreover, epitranscriptomic regulation in brain development and function also has drawn attention, in particular N^6^-methyladenosine (m^6^A) modification [19–21].

As the most important form of RNA methylation, m^6^A modification plays a vital role in regulating gene expression, protein translation, and cell behaviors [22–26]. m^6^A modification is exceled through a coordination of three types of enzymes: (i) methyltransferase complex, also called “writers,” for example Mettl3, Mettl14, and Wtap, is responsible for methylation of RNA transcripts at an appropriate site; (ii) m^6^A-binding proteins, called “readers,” recognize m^6^A sites in RNAs to promote RNA maturation and translation; (iii) demethylases, named “erasers,” for instance Fto and Alkbh5, are responsible for removing m^6^A modification from RNAs [21, 27, 28]. Tight regulations of methyltransferase and demethylase are crucial for brain development [19, 27–31]. Although m^6^A modification does not change nucleic acid base pairing, its dysregulation alters essential biological processes, and is associated with various diseases such as neurological disorders and cancers [32–36]. Moreover, mechanistic studies have shown that methyltransferase is involved in transcriptional and translational regulations of downstream genes [24, 35, 37–39]. How methyltransferase and demethylase regulate expression of downstream genes during brain development remains unclear.

To distinguish biological functions of Fto and Mettl3 in brain development, we individually created *Fto* and *Mettl3* cortical-specific conditional knockout mice. Knockout of *Mettl3* causes more severe cortical defects than deletion of *Fto*. RNA sequencing (RNA-seq) and ribosome profiling sequencing (Ribo-seq) uncover that both Mettl3 and Fto regulate gene expression at transcriptional and translational levels. Interestingly, deletion of *Mettl3* has a more severe impact on proper ribosome distribution than knockout of *Fto*, causes elevated translation of a significant number of mRNAs, and leads to a more severe disruption of biological functions of mRNAs at the translational level. Our study provides new evidence of translational regulations of Mettl3, and demonstrates distinct roles of *Mettl3* and *Fto* in cortical development.

## Results

### Cortical-specific deletion of *Fto* has mild effects on brain development

As an m^6^A demethylase, whether Fto is required for proper development of the cerebral cortex remains unknown. To investigate the role of Fto in cortical development, we constructed *Fto* conditional knockout mice, referred as *F*-cKO, by breeding floxed *Fto* mice with *Emx1*-Cre mice, in which Cre recombinase is expressed under the *Emx1* promoter that is active only in the cerebral cortex [40] (Supplementary Fig. S1A). We verified and confirmed deletion of the exon 3, which is ablated in our knockout strategy, in cortical tissues of embryonic day 13.5 (E13.5) *F*-cKO mice using reverse transcription PCR (RT-PCR) (Supplementary Fig. S1B).

Newborn *F*-cKO mice survived well and did not show obvious changes in body size and body weight, compared to their wild-type control (Ctrl) littermates at postnatal day 14 (P14) (Supplementary Fig. S1C, D). We did not observe detectable changes in brain size in *F*-cKO mice, compared to their control littermates (Supplementary Fig. S1E, F). However, there was a slight decline in survival rate (80% of controls) in *F*-cKO mice by P20 (Supplementary Fig. S1G). These results indicate that Fto cortical deletion has mild effects on survival and brain development in mice.

**Fig. 1:**
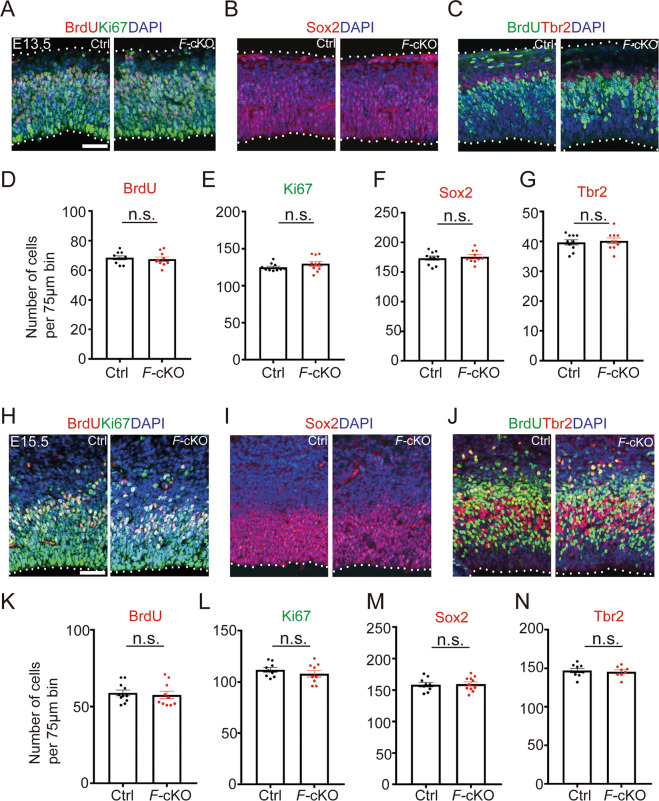
Deletion of *Fto* in the cerebral cortex does not change development of neural progenitors. **A**–**G** Immunohistochemistry (**A**–**C**) and cell counting (**D**–**G**) in the cerebral cortex of E13.5 *F*-cKO mice. The numbers of BrdU^+^ and Ki67^+^ cells, and Sox2^+^ and Tbr2^+^ neural progenitors were not changed in E13.5 *F*-cKO cortices, compared to controls (Ctrl). **H**–**N** Immunohistochemistry (**H**–**J**) and cell counting (**K**–**N**) in the cerebral cortex of E15.5 *F*-cKO mice. The numbers of BrdU^+^ and Ki67^+^ cells, and Sox2^+^ and Tbr2^+^ neural progenitors were not changed in E15.5 *F*-cKO cortices, compared to the Ctrl. Scale bars: 25 μm. Error bars indicate the SEM (six independent samples). *P* values were calculated by Student’s *t*-test between Ctrl and *F*-cKO. *P* values: n.s.: non-significant.

We then examined cortical development in the *F*-cKO mouse. We collected mouse brain tissues and examined development of neural progenitors and newborn neurons in E13.5 and E15.5 cortices. We used an anti-bromodeoxyuridine (BrdU) antibody to label neural progenitors in the S-phase after 1 h BrdU pulse before tissue collection, and an anti-Ki67 antibody to label those in the G1, S, G2, and M phase in a cell cycle (Fig. 1A, H). In E13.5 and E15.5 cortices, numbers of BrdU^+^ and Ki67^+^ cells were compatible between *F*-cKO and control mice (Fig. 1D, E, K, L).

We next examined radial glial cells (RGCs) and intermediate progenitors (IPs) by labeling anti-Sox2 and anti-Tbr2 antibodies, respectively (Fig. 1B, C, I, J). We also did not detect differences in the number of Sox2^+^ and Tbr2^+^ neural progenitors in E13.5 and E15.5 (Fig. 1F, G, M, N). Moreover, we labeled deep-layer and upper-layer neurons in the cortex using anti-Tbr1 and -Satb2 antibodies, respectively. The numbers of Tbr1^+^ and Satb2^+^ neurons showed no significant difference between *F*-cKO and control cortices at P0 and P14 (Supplementary Fig. S2A–H). We also used anti-NeuN antibodies to mark mature neurons and did not detect significant changes in numbers of NeuN^+^ neurons in P0 and P14 *F*-cKO cortices, compared to those in controls (Supplementary Fig. S2I–L). These results indicate that cortical-specific *Fto* knockout does not affect proliferation of neural progenitors and production of newborn neurons in the cortex.

### *Mettl3* conditional knockout mice show brain defects

Previous studies have shown that both methyltransferase and demethylase are involved in regulating m^6^A status [27, 41]. Because Mettl3 has the core of catalytic activity of the methyltransferase complex, and deletion of its binding partner *Mettl14* displays brain defects [19, 42, 43], we predicted that Mettl3 is crucial for cortical development. We generated cortical-specific *Mettl3* conditional knockout mice, named *M*-cKO, by breeding floxed *Mettl3* mice with *Emx1*-Cre mice (Supplementary Fig. S3A). RT-PCR verification also indicated successful deletion of the exon 2 and 3 in E13.5 *M*-cKO cortices (Supplementary Fig. S3B). Noticeably, newborn *M*-cKO mice looked smaller, and P14 pups showed significant weight loss, compared to control littermates (Supplementary Fig. S3C, D). Dead *M*-cKO pups were observed at P14, and the survival rate of *M*-cKO mice was significantly declined at P20, and reached 30% by P30, compared to their control littermates, suggesting an early lethality of *M*-cKO mice (Supplementary Fig. S3E).

We then examined brain size, measured lengths of the cortex and brain at P14, and found that the cortical size is significantly reduced in *M*-cKO mice, even though the whole brain also was smaller, compared to those in controls (Supplementary Fig. S3F–H). These results indicate that the cortical size is more profoundly reduced, compared to the whole brain size, due to knockout of *Mettl3*.

### Knockout of *Mettl3* causes increased number of intermediate progenitors

To understand causes of smaller cortices in *M*-cKO mice, we first examined progenitor development. We did not detect obvious changes in the number of BrdU^*+*^ and Ki67^*+*^ proliferative cells in E13.5 control and *M*-cKO cortices (Supplementary Fig. S4A–C). Numbers of Sox2^+^ and Tbr2^+^ neural progenitors also did not display differences between two groups (Supplementary Fig. S4D–F). These results suggested a normal early development of neural progenitors in *M-*cKO cortices.

**Fig. 2:**
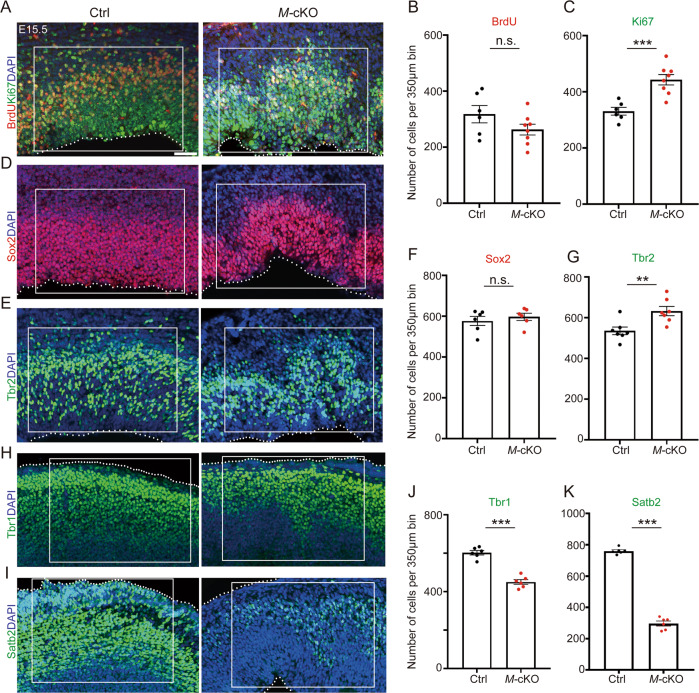
Cortical-specific deletion of *Mettl3* causes neurogenesis defects in E15.5 cortex. **A**–**C** The number of BrdU^+^ cells (red) was not changed, the number of Ki67^+^ cells (green) was increased in *M*-cKO cortices, compared to controls (Ctrl). **D**–**G** The number of Sox2^+^ neural progenitors was not changed, the number of Tbr2^+^ neural progenitors was increased in *M*-cKO cortices, compared to the Ctrl. **H**–**K** The numbers of Tbr1^+^ and Satb2^+^ neurons were reduced in *M*-cKO cortices, compared to the Ctrl. Scale bars: 70 μm. Error bars indicate the SEM (six independent samples). *P* values were calculated by Student’s *t*-test between Ctrl and *M*-cKO. *P* value: **P < 0.01; ***P < 0.001; n.s.: non-significant.

Interestingly, we observed folding structures in the cortex, in particular in the VZ and SVZ, in coronal sections of E15.5 *M*-cKO brains after Nissl staining (Supplementary Fig. S4G). We found that the number of BrdU^+^ cells is not changed; on the contrary, the number of Ki67^+^ cells is significantly increased in E15.5 *M*-cKO cortices, suggesting an elevated number in proliferative cells due to *Mettl3* knockout (Fig. 2A–C). Moreover, while the numbers of Sox2^+^ and Pax6^+^ progenitors was not altered, the number of Tbr2^+^ progenitors was greatly increased in E15.5 *M*-cKO cortex, compared to those in the control (Fig. 2D–G and Supplementary Fig. S5C, D). We then examined the number of Sox2^+^BrdU^+^ cells and did not detect significant changes, suggesting that the number of dividing RGCs is not affected (Supplementary Fig. S5A, B). We also tested the number of Sox2^+^Tbr2^+^ cells and did not find obvious changes, suggesting that the number of transition cells from RGCs to IPs is not altered (Supplementary Fig. S5E, F). Finally, we quantified the number of Tbr2^+^BrdU^+^ cells, and found that the number of Tbr2^+^BrdU^+^ cells is increased in *M*-cKO cortices, indicating an increased dividing IP population (Supplementary Fig. S5G, H).

These results indicate that *Mettl3* knockout causes a progressive increase in numbers of neural progenitors, and results in expansion of IPs but not RGCs.

### Deletion of *Mettl3* inhibits neuronal production

Because numbers of neural progenitors are increased due to *Mettl3* knockout, we next investigated whether cortical neuronal production also is affected. We first examined the Tbr1^+^ deep-layer neurons at an early stage, and found that the number of Tbr1^+^ cells is significantly decreased in the E13.5 *M*-cKO cortex, suggesting an early defect of neuronal production due to Mettl3 deletion (Supplementary Fig. S6A, B). Moreover, similar to folding structures detected in the VZ and SVZ, CP also was folded in E15.5 *M*-cKO cortices (Fig. 2H, I). We found that the number of Tbr1^+^ and Satb2^+^ cells is decreased in the *M*-cKO cortex, compared to that in the control, suggesting reduced differentiation of newborn neurons (Fig. 2J, K). In addition, folding structures in the CP were undetectable in P0 and P14 *M*-cKO cortices (Supplementary Fig. S7). However, numbers of Tbr1^+^, Satb2^+^, and NeuN^+^ cells remained significant reduction in P0 and P14 *M*-cKO cortices (Supplementary Fig. S7).

To test whether apoptosis contributed to reduced neurogenesis, we examined the number of Casp3^+^ cells, which represent those undergoing apoptosis. We detected an increased Casp3^+^ cells in cortices in the *M*-cKO, suggesting an elevated apoptosis due to Mettl3 knockout (Supplementary Fig. S6C–F).

These results indicate that *Mettl3* knockout causes reduced neuronal differentiation and production, and promotes apoptosis in cortical cells, which might lead to a smaller cortex.

### Profound alterations of RNA transcriptome in *Mettl3* knockout cortices

**Fig. 3:**
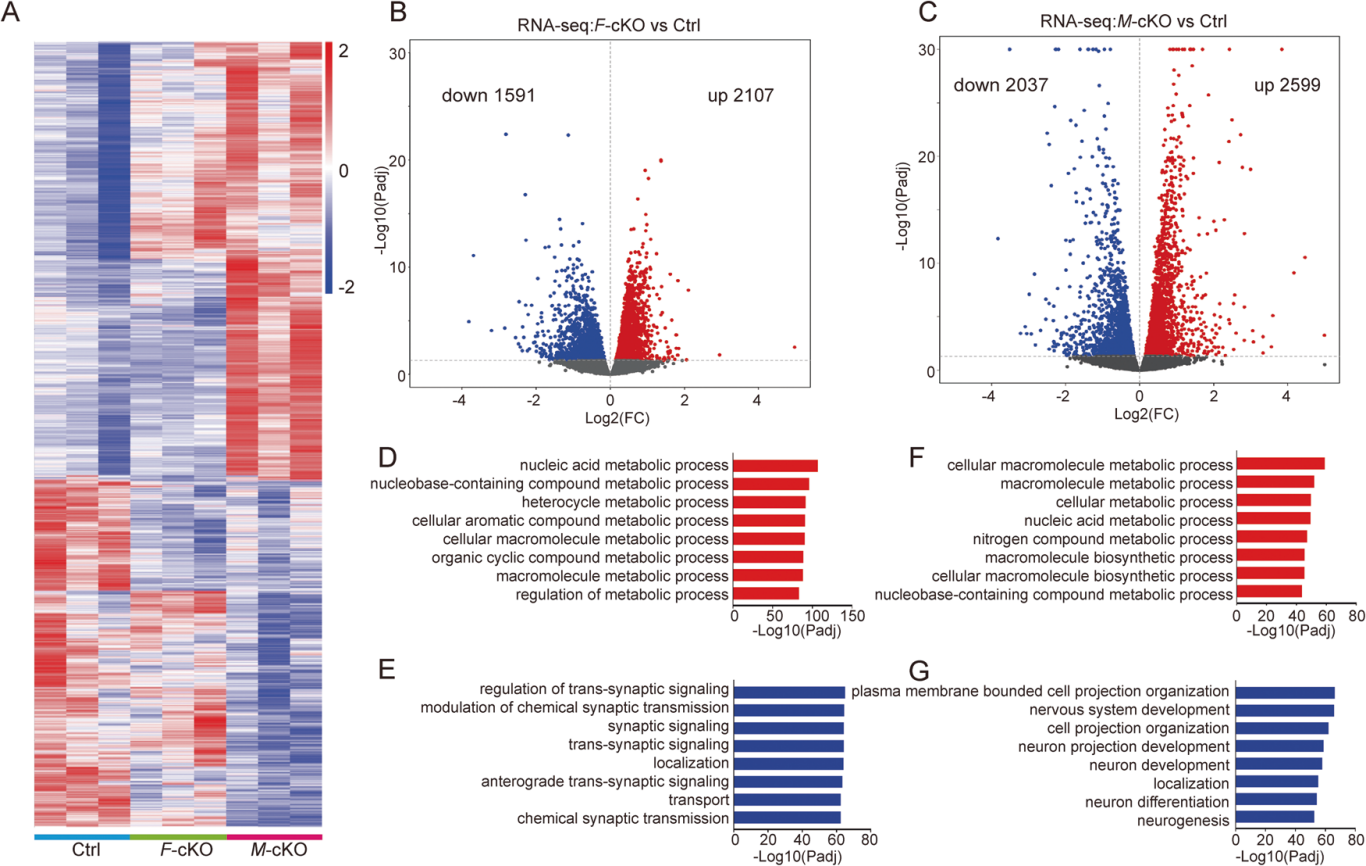
*Mettl3* knockout causes more profound transcriptomic alterations than *Fto* deletion. **A** Heat map of differentially expressed genes in E15.5 control (Ctrl), *F*-cKO and *M*-cKO cortices detected by RNA sequencing (RNA-seq). **B**, **C** Volcano map of up- and downregulated genes in *M*-cKO (**B**) and *F*-cKO (**C**) cortices compared to the Ctrl. More genes were upregulated and downregulated in *M*-cKO than in *F*-cKO. **D**, **E** Gene Ontology (GO) analysis of upregulated genes (**D**) and downregulated genes (**E**) in *M*-cKO cortices. **F**, **G** GO analysis of upregulated genes (**F**) and downregulated genes (**G**) in *M*-cKO cortices.

To investigate a reasoning of severe cortical defects in *M*-cKO and mild phenotypes in *F*-cKO mice, we examined RNA transcription profiles in E15.5 *M*-cKO and *F*-cKO mouse cortices using RNA-seq analyses with three biological replications from each mouse line. RNA-seq data among control and knockout cortical replicate samples were highly consistent as detected using principal component analysis and Pearson correlation coefficient analysis (Supplementary Fig. S8A, B). Even though RNA transcriptional levels showed significant changes in both *F*-cKO and *M*-cKO cortices, more differentially expressed RNA transcripts were detected in the *M*-cKO cortex than those in the *F*-cKO group, compared to the control cortex (Fig. 3A and Supplementary Table S1). Compared to controls, there were 2107 RNA transcripts upregulated and 1591 downregulated in *F*-cKO cortices (Fig. 3B and Supplementary Table S2), and 2599 RNA transcripts upregulated and 2037 downregulated in *M*-cKO cortices (Fig. 3C and Supplementary Table S3). These results suggest that knockout of *Mettl3* has a more dramatic effect on gene transcription than knockout of *Fto*.

We next performed Gene Ontology (GO) analyses on differentially expressed RNA transcripts in *F*-cKO and *M*-cKO cortices (Fig. 3D–G and Supplementary Tables S4–S7). We found that upregulated genes are mainly concentrated in metabolic-related items in both *M*-cKO and *F*-cKO cortices, while downregulated genes are associated with transport, and synaptic signaling in *F*-cKO, and in nervous system development, and neurogenesis in *M*-cKO (Fig. 3D–G). These results suggest that severe cortical defects due to *Mettl3* knockout are likely caused by alterations of RNA transcripts that normally play fundamental roles in neurogenesis.

### Ribo-seq reveals severely altered translational regulation in *Mettl3* knockout cortices

Recent studies have shown that Mettl3 plays a role in regulating translational control [35, 37]. Taking a consideration of severe defects in *M*-cKO cortices, we suspected that altered RNA transcriptome alone might not explain all these phenotypes. Thus, we quantified abundance of ribosome-protected fragments (RPFs) that are undergoing translation in real time at a genome-wide level by conducting Ribo-seq in E15.5 control, *M*-cKO, and *F*-cKO cortices.

**Fig. 4:**
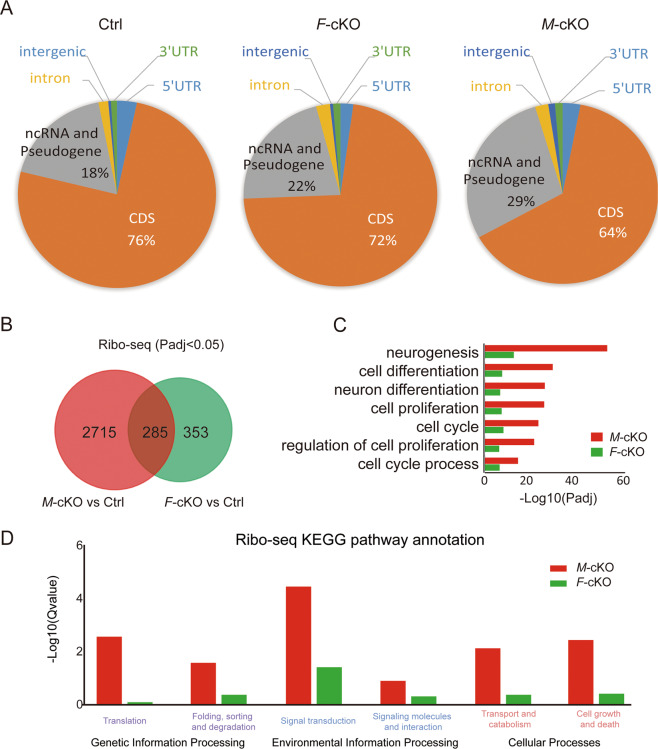
*Mettl3* knockout results in more severe translational dysregulation than *Fto* deletion. **A** Pie charts of ribosome distributions in coding sequence (CDS) of mRNA transcripts, noncoding RNAs (ncRNAs), and pseudogenes in E15.5 control (Ctrl), *F*-cKO, and *M*-cKO cortices detected by ribosome profiling sequencing (Ribo-seq). **B** Venn diagram of differentially expressed mRNAs between *M*-cKO and *F*-cKO. **C** Gene Ontology (GO) enrichment analyses of differentially expressed mRNAs between *M*-cKO and *F*-cKO. **D** Kyoto Encyclopedia of Genes and Genomes (KEGG) pathway analyses of mRNAs that show significantly differential expression (*P*_adj_ < 0.05) in *M*-cKO (Red) and *F*-cKO (green) cortices.

As predicted, Ribo-seq recognized 28 nucleotide (nt) ribosome footprints, which were mainly mapped in coding sequence (CDS) of mRNA transcripts, with few mapped in noncoding RNAs (ncRNAs) and pseudogenes (Supplementary Fig. S8C, D). Interestingly, while 76% and 72% ribosome footprints were mapped in the CDS in control and *F*-cKO cortices, respectively, only 64% mapped in the CDS and 29% mapped in ncRNAs and pseudogenes in *M*-cKO cortices (Fig. 4A). The dramatic reduction of ribosome footprints in CDS of mRNAs in *M*-cKO cortices suggests that knockout of *Mettl3* has a more severe impact on proper ribosome distribution than deletion of *Fto*.

Next, we analyzed numbers of ribosome-covered mRNAs that also displayed differential expression (*P*_adj_ < 0.05) in *M*-cKO and *F*-cKO cortices, as compared with those in controls. Surprisingly, while 3000 differential mRNAs were detected in *M*-cKO cortices, only 638 mRNAs were observed in *F*-cKO cortices, which was 21% of those in *M*-cKO cortices (Fig. 4B and Supplementary Table S8). And 285 differential mRNAs were detected in both *M*-cKO and *F*-cKO cortices. These results indicate that knockout of *Mettl3* causes a more severe disruption of translational regulation of mRNAs than deletion of *Fto*. Furthermore, we performed GO analyses on differential mRNAs, and found that a significantly greater number of differential mRNAs was enriched in terms of regulations in cell differentiation, cell cycle, and proliferation in *M*-cKO cortices than those in *F*-cKO (Fig. 4C). These results further support a more severe disruption of biological functions at the translational level due to *Mettl3* knockout.

We then conducted Kyoto Encyclopedia of Genes and Genomes (KEGG) analyses, and detected a significant number of altered mRNAs annotated in pathways such as translation, folding, cell growth, and death in *M*-cKO cortices, compared to those in controls (Fig. 4D and Supplementary Table S9). These KEGG analyses suggest that there are more altered mRNAs involved in essential biological pathways in *M*-cKO cortices than those in *F*-cKO, which further supports severe functional alterations caused by *Mettl3* knockout.

### Altered translation efficiency (TE) caused by *Mettl3* and *Fto* knockout

Because knocking out *Mettl3* and *Fto* altered both transcriptional and translational regulations, we next examined whether translation of some mRNAs is specifically suppressed or elevated by analyzing TE, which can be calculated by comparing corresponding sequencing abundances of each mRNA detected by both Ribo-seq and RNA-seq.

**Fig. 5:**
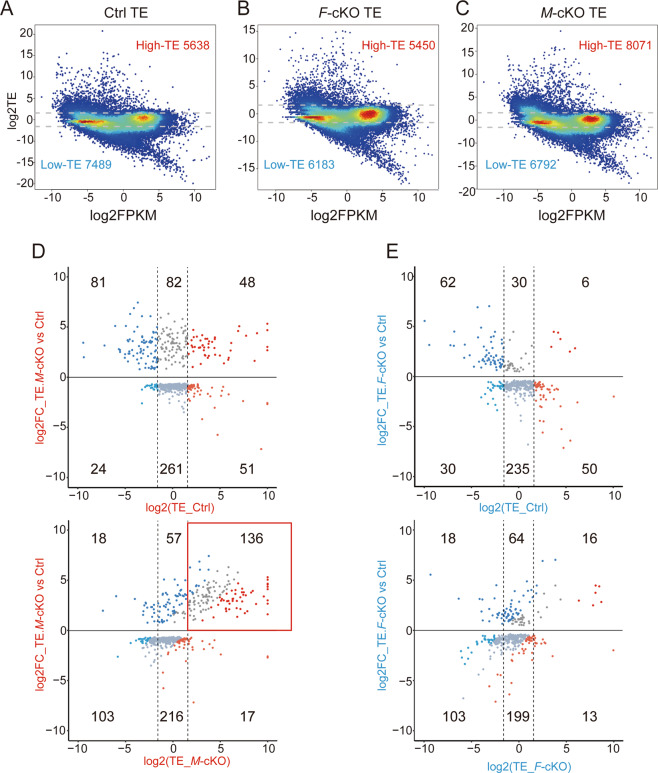
Altered translation efficiency (TE) caused by *Mettl3* and *Fto* knockout. **A**–**C** Numbers of mRNAs with low-TE and high-TE in E15.5 control (Ctrl), *F*-cKO, and *M*-cKO cortices detected by Ribo-seq and RNA-seq. **D**, **E** Distributions of significantly differentially expressed mRNAs in three intervals of high-, medium-, and low-TE in the six-quadrant diagram. The red box illustrates mRNAs with most significantly altered TE.

We identified 5638 mRNAs with high-TE and 7489 mRNAs with low-TE in the control cortex, using criteria that the TE for an mRNA is significantly lower or higher than the median, and the difference is at least threefold (Fig. 5A–C and Supplementary Table S10) [44]. While 5450 mRNAs with high-TE and 6183 mRNAs with low-TE were detected in the *F*-cKO cortex, 8071 mRNAs with high-TE and 6792 mRNAs with low-TE were identified in the *M*-cKO cortex (Fig. 5A–C). More than 2433 and 697 mRNAs displayed high-TE and low-TE, respectively, in the *M*-cKO cortex compared to those in the control. These results suggest that knockout of *Mettl3* causes elevated translation of a significant number of mRNAs, and deletion of *Fto* results in translational suppression of some mRNAs.

We then used a six-quadrant diagram to further analyze distributions of significantly differentially expressed mRNAs in three intervals of high-, medium-, and low-TE. We did not observe obvious changes of distributions of mRNAs in the *F*-cKO cortex in the six-quadrant diagram, we detected a significant shift of mRNAs into the high-TE quadrant in the *M*-cKO cortex (Fig. 5D, E). These results suggest that deletion of *Fto* causes subtle changes of TE for most mRNAs, while knockout of *Mettl3* causes elevated TE for many mRNAs.

### Deletion of *Mettl3* and *Fto* leads to altered expression of major genes expressed in RGCs and IPs

Because neural progenitor defects were detected in *M*-cKO but not in *F*-cKO cortices, we focused on examining transcriptional and translational status of genes highly expressed in RGCs and IPs. We identified 471 RGC-expressing and 322 IP-expressing genes, based on previous reports of mouse cortical transcriptome analyses (Supplementary Tables S11 and S12) [45, 46]. Among 471 RGC genes, 227 genes were upregulated and 39 were downregulated in *F*-cKO cortices, and 163 genes were upregulated and 61 were downregulated in *M*-cKO cortices (Supplementary Fig. S9A). Moreover, GO enrichment analyses showed that upregulated genes are associated with cell cycle regulations in both *M*-cKO and *F*-cKO cortices (Supplementary Fig. S9C). Interestingly, downregulated transcripts were associated with organism development in *F*-cKO cortices, and with cell development, and neurogenesis in *M*-cKO cortices (Supplementary Fig. S9C). These results suggest that downregulation of genes involved in neurogenesis in RGCs contributes to cortical defects when *Mettl3* is deleted.

In addition, 145 IP-expressing genes were upregulated and 18 downregulated in *F*-cKO cortices, and 108 IP-expressing genes were upregulated and 38 downregulated in *M*-cKO (Supplementary Fig. S9B). GO analyses showed terms of cell cycle process for upregulated IP genes in *F*-cKO and *M*-cKO cortices, and terms of regulation of monocyte chemotaxis in *F*-cKO, and cellular component organization or biogenesis in *M*-cKO cortices for downregulated IP genes (Supplementary Fig. S9D). These results suggest that altered cell cycle regulation in upregulated IP genes due to *Mettl3* deletion is associated with cortical defects in *M*-cKO mice.

**Fig. 6:**
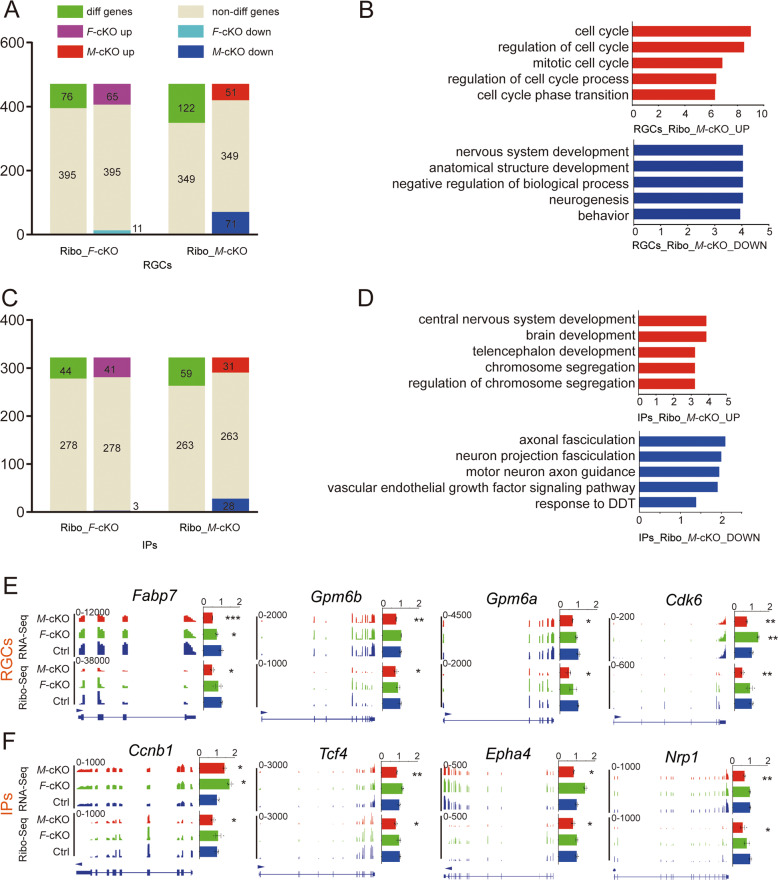
Altered expression of major genes expressed in RGCs and IPs in *M*-cKO and *F*-cKO cortices. **A** Numbers of RGC genes showed different (diff genes), none different (non-diff genes), up- and downexpression in *F*-cKO and *M*-cKO cortices detected by Ribo-seq. **B** GO analyses of upregulated (red) and downregulated (blue) RGC genes in *M*-cKO cortices. **C** Numbers of IP genes showed different, none different, up- and downexpression in *F*-cKO and *M*-cKO cortices detected by Ribo-seq. **D** GO analyses of upregulated (red) and downregulated (blue) RGC genes in *M*-cKO cortices. **E**, **F** Integrative Genomics Viewer (IGV) images of expression patterns (left) and expression quantifications (right) of *Fabp7*, *Gpm6b*, *Gpm6a*, *Cdk6* (E), and *Ccnb1*, *Tcf4*, *Epha4*, *Nrp1* (F) in E15.5 control (Ctrl), *F*-cKO, and *M*-cKO cortices detected by RNA-seq and Ribo-seq. Error bars indicate the SEM (three independent samples). *P* values were calculated by Student’s *t*-test between Ctrl and *F*-cKO or Ctrl and *M*-cKO. *P* values: *P < 0.05; **P < 0.01; ***P < 0.001.

We next analyzed translational status in RGCs and IPs using dataset in Ribo-Seq. Among 471 RGC genes, 65 genes were upregulated and 11 were downregulated in *F*-cKO cortices, and 51 genes were upregulated and 71 were downregulated in *M*-cKO cortices (Fig. 6A). GO enrichment analysis showed that upregulated genes in RGCs in *F*-cKO and *M*-cKO cortices were associated with cell cycle, and downregulated genes in RGCs in *M*-cKO cortices were enriched in the anatomical structure development, negative regulation of biological process, and neurogenesis (Fig. 6B and Supplementary Fig. S9E).

Moreover, 41 IP-expressing genes were upregulated and 3 downregulated in *F*-cKO, and 31 IP-expressing genes were upregulated and 28 downregulated in *M*-cKO cortices (Fig. 6C). GO analyses showed enriched terms of mitotic sister chromatid segregation and mitotic nuclear division of upregulated IP genes in *F*-cKO cortices, upregulated genes associated with central nervous system development, and downregulated genes associated with axonal fasciculation, and neuron projection fasciculation in *M*-cKO cortices (Fig. 6D and Supplementary Fig. S9F). These results suggest that Mettl3 deficiency causes altered translation of RGC- and IP-expressing genes associated with neurogenesis, axonal guidance, and neuronal projection.

Furthermore, we illustrated expression patterns of some RGC- and IP-expressing genes using Integrative Genomics Viewer (IGV). We quantified expression of these mRNAs using RPKM based on RNA-seq and Ribo-seq data, and normalized their average values in *M*-cKO and *F*-cKO cortices with those in the control cortex. As detected by Ribo-Seq, we found that RGC-expressing genes Fabp7, Gpm6a, Gpm6b, and Cdk6 show significant downregulation in the *M*-cKO, but not in *F*-cKO cortices, when compared with those in the control cortex (Fig. 6E). IP-expressing genes Ccnb1, Tcf4, Epha4, and Nrp1 were downregulated in *M*-cKO, but not in *F*-cKO cortices (Fig. 6F). These analyses further suggest that altered translation of crucial genes in RGCs and IPs, due to deletion of Mettl3, may disrupt proliferation and differentiation of neural progenitors and cause abnormal cortical development.

### Knockout of *Mettl3* alters expression levels of major components in m^6^A methylation

**Fig. 7:**
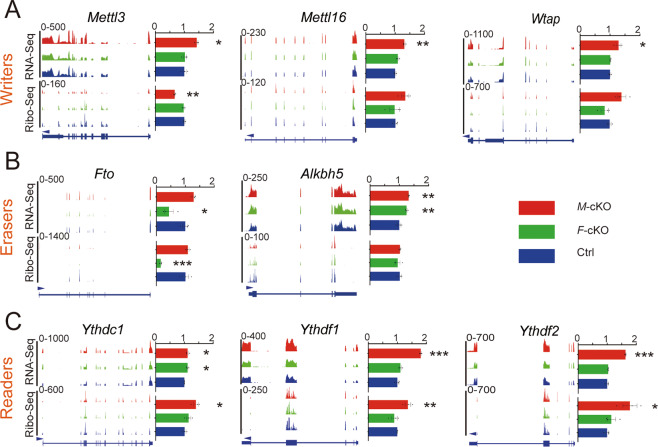
Knockout of *Mettl3* alters expression levels of major components in m^6^A methylation. **A** Integrative Genomics Viewer (IGV) images of expression patterns (left) and expression quantifications (right) of *Mettl3*, *Mettl16*, and *Wtap* in E15.5 control (Ctrl), *F*-cKO, and *M*-cKO cortices detected by RNA-seq and Ribo-seq. **B** Expression patterns (left) and quantifications (right) of demethylases of *Fto* and *Alkbh5*. **C** Expression patterns (left) and quantifications (right) of *Ythdc1*, *Ythdf1*, and *Ythdf2*. Error bars indicate the SEM (three independent samples). *P* values were calculated by Student’s *t*-test between Ctrl and *F*-cKO or Ctrl and *M*-cKO. *P* values: *P < 0.05; **P < 0.01; ***P < 0.001.

Because Mettl3 and Fto are key components of m^6^A methylation, we predicted that knockout of *Mettl3* and *Fto* might affect expression of other members of “writers,” “readers,” and “erasers.” We illustrated expression patterns of major components in m^6^A methylation using IGV, and quantified expression of these mRNAs using RPKM based on RNA-seq and Ribo-seq data, and normalized their average values in *M*-cKO and *F*-cKO cortices with those in the control cortex (Supplementary Table S13). We found that translational levels of Mettl3 and Fto as detected by Ribo-seq are significantly reduced in *M*-cKO and *F*-cKO cortices, respectively, suggesting a successful knockout of *Mettl3* and *Fto* (Fig. 7A, B).

Interestingly, we found that expression levels of most methyltransferases, in particular Mettl16, Wtap, and Rbm15, are dramatically elevated as detected by both RNA-seq and Ribo-seq in *M-*cKO cortices, but they are not significantly changed in *F-*cKO cortices (Fig. 7A and Supplementary Fig. S8E). These results suggest that *Mettl3* deletion might lead to a compensation effect of other methyltransferases. Moreover, while knockout of *Mettl3* and *Fto* caused increased expression of “eraser” *Alkbh5* as detected by RNA-seq, it did not alter Alkbh5 expression at the protein level (Fig. 7B). In addition, expression levels of most “readers” such as Prrc2a, Ythdf1, Ythdf2, and Ythdc1 also were upregulated in *M-*cKO but not in *F*-cKO cortices, in particular as detected by Ribo-seq (Fig. 7C and Supplementary Fig. S8E). These results further suggest that knockout of *Mettl3* but not *Fto* has a profound effect on “reader” proteins in m^6^A modifications, and might in turn alter mRNA maturation and translation.

## Discussion

Increasing evidence has demonstrated the importance of epitranscriptomic regulation in brain development and function, in particular m^6^A methylation. Whether RNA methyltransferases and demethylases function distinctly in the cerebral cortex remains unclear. In this study, we demonstrate that Mettl3 plays a more profound role in cortical development than Fto does using mouse genetic tools. We show that knockout of *Mettl3* has a more severe impact on gene transcriptional and translational regulation of mRNAs based on RNA-seq and Ribo-seq analyses. Our results indicate crucial and distinct roles of Mettl3 and Fto in cortical development, and highlight Mettl3 regulation on major neural mRNAs through translational regulations in the cortex.

Gene knockout and knockdown approaches have been used to study biological functions of key components of methyltransferases. Deletion of *Mettl14* leads to prolonged cortical neurogenesis to the postnatal stage [19]. *Mettl14* deletion in the striatum results in increased neuronal excitability and impaired striatal-mediated behaviors [47]. Loss of *Mettl14* or m^6^A-binding protein *Ythdf1* causes decreased injury-induced protein translation in adult dorsal root ganglions and functional axon regeneration in the peripheral nervous system [48]. Moreover, both Mettl3 and Mettl14 have the domain of methyltransferase. However, only Mettl3 has the catalytic activity, suggesting its crucial biochemical role [49]. Indeed, we have found that cortical-specific knockout of *Mettl3* causes increased expansion of IPs, and reduced neuronal production, which leads to folding structures in the cortex. Mechanistically, *Mettl3* depletion causes RNA aberrant splicing events and dysregulation of transcriptome-wide gene expression [50]. Consistent with previous studies, we have found that cortical deletion of *Mettl3* causes large-scale RNA transcriptomic changes, which might contribute to severe brain phenotypes. Moreover, we have observed reduced translational level of Mettl3 as detected by Ribo-seq, and increased expression of Mettl3 mRNA as detected by RNA-seq. We think this might be due to the gene knockout strategy for the *Mettl3* gene. Nevertheless, studies from others and us demonstrate that loss of either Mettl3 or Mettl14 leads to severe biological defects, indicating equally crucial roles of Mettl3 and Mettl14, and a non-compensating effect between them.

Genetic approaches have also been used to study functions of demethylase such as Fto. *Fto* knockout mice show postnatal growth retardation and a lean body mass [51]. In the nervous system, Fto is expressed in the brain, and *Fto* knockout causes reduced proliferation of adult neural stem cells in hippocampus, and leads to impaired learning and memory [52, 53]. Surprisingly, we have found that cortical-specific knockout of *Fto* does not cause detectable phenotypes in the embryonic brain. One possible reason is that Fto function in the brain might be compensated by other demethylases such as Alkbh5, as we have found that *Alkbh5* transcription level is upregulated in the *Fto* knockout cortex. The future work is to study how demethylases work cooperatively and control brain development and function.

In addition, previous studies indicate that a dynamic and reversible modification of m^6^A is conducted by three elements: “writers,” “readers,” and “erasers.” “Writers” such as Mettl3 are responsible for placing m^6^A modifications on primary mRNA (pri-mRNA) [41, 54–56]. And the modified RNA is recognized and processed by “readers” for instance HnrnpC to facilitate RNA maturation [57–62]. Moreover, m^6^A can be eliminated by “erasers” such as Fto [27, 28]. It is possible that “erasers” work effectively only under a circumstance when m^6^A modification occurs at a wrong site or a wrong time in a pri-mRNA, in which “erasers” function in a default correction manner. This might explain why cortical-specific knockout of *Fto* shows subtle brain defects, while *Mettl3* conditional knockout displays severe phenotypes. Furthermore, previous studies have shown that the level of N6, 2-Odimethyladenosine (m^6^Am) is increased when Fto is knocked out, suggesting that Fto perhaps primarily targets m^6^Am but not m^6^A [63, 64]. These results suggest that interactions between Mettl3 and Fto may be not a simple antagonistic or cooperative mode.

Notably, besides functioning in m^6^A modification, methyltransferases such as Mettl3 and Mettl14 play regulatory roles in transcriptional and translational regulations. Studies have shown that METTL3, independently of METTL14, can be directly recruited to the transcriptional start sites of active genes, suggesting its role in transcriptional regulation [37]. METTL14 regulates stability of target transcripts through RNA stability factor HuR [36]. Knocking out *Mettl14* in the central nervous system results in alterations of transcription levels of a broad range of target genes, which might be a direct and indirect outcome [19]. Moreover, Mettl3 function in modulating translation has been highly appreciated. For instance, METTL3 appears to enhance translation of its binding RNA transcripts by relieving ribosome stalling in cancer cells [37]. In addition, METTL3 promotes translation of a large subset of oncogenic mRNAs through directly interacting with the eukaryotic translation initiation factor 3 subunit h [39]. These results highlight the importance of methyltransferases, in particular Mettl3, in regulating transcription and translation, and in turn affect cell behaviors.

Our study has provided new evidence of regulatory roles of Mettl3 and Fto in modifying transcription and translation of cortical mRNAs, based on dataset of RNA-seq and Ribo-seq. Knockout of *Mettl3* appears to have a more profound effect on a large number of transcripts, in particular those functions in cell cycle regulation and neurogenesis, compared to deletion of *Fto*. Our findings indicate that knockout of *Mettl3* disrupts translational levels of more progenitor genes than deletion of *Fto*, which may cause more severe phenotypes in the *M*-cKO cortex. Therefore, our RNA-seq and Ribo-seq data have provided molecular evidence that interprets the more severe brain defects in *Mettl3* knockout mice compared to those in *Fto* knockout. The future study should be to uncover biochemical details of how Mettl3 and Fto distinctly regulate transcription and translation. In particular, the direct causality between Mettl3 and translational regulation requires further biochemical studies to decipher whether it is due to translational regulation itself or a side-product of altered mRNA decay.

In summary, we have found that RNA methyltransferases and demethylases function distinctly in cortical development. In the cerebral cortex, Mettl3 plays a more profound role in controlling the size of neural progenitor pool, neuronal production, and transcription and translation of major neural genes than Fto does. Our study highlights the complexity of m^6^A methylation in regulating downstream genes, and in turn excel its biological function.

## Materials and methods

### Construction of floxed *Fto* and *Mettl3* transgenic mice

Homologous recombination was used, cas9 mRNA, gRNA, and donor vector were microinjected into the fertilized eggs of C57BL/6J mice to obtain F0 generation mice. Floxed *Fto* and *Mettl3* heterozygous mice showed no obvious abnormalities.

### Generation of *Fto* and *Mettl3* cortical-specific conditional knockout mice

Floxed *Fto* and *Mettl3* transgenic mice (C57/BL6 × 129 background) were bred with *Emx1-Cre* mice (C57/BL6 background) to generate *F*-cKO and *M*-cKO mice.

For staging of embryos, midday of the day of vaginal plug formation was considered embryonic day 0.5 (E0.5), and the first 24 h after birth was defined as postnatal day 0 (P0). Animal use was overseen by the animal facility at the Huaqiao University, and was performed according to the institutional ethical guidelines for animal experiments.

Mouse tail tip biopsies were used for genotyping by PCR using the following primers: *Cre*, 5’-TAAAGATATCTCACGTACTGACGGTG-3’ and 5’-TCTCTGACCAGAGTCATCCTTAGC-3’ (product size: 300 bp); *Mettl3*, 5’-ACACGCTCACATTCCTTAGTTAC-3’ and 5’-TCTGTTTTCTGAATCCTCTTTTG-3’ (product sizes: 434 bp from the floxed allele and 376 bp from the wild-type allele); *Fto*, 5’-AGAGTACCTGAATGCTTCAGGGTT-3’ and 5’-ATCTTCAACACAAACAAAAAAATC-3’ (product sizes: 300 bp from the floxed allele and 255 bp from the wild-type allele).

### Reverse transcription PCR (RT-PCR)

Total RNA was isolated using TRIzol LS Reagent (Life Technologies, USA). One microgram total RNA was used for reverse transcription using High-Capacity Cdna Reverse Transcription Kit (ThermoFisher, Waltham, MA). Primers used in this study were as following.

*M*-cKO-Exon1: 5’-TTCATCTTGGCTCTATCCGGC-3’; 5’-ATAGAGCTCCACGTGTCCGA-3’; *M*-cKO-Exon2-3: 5’-TCTCAGATCTGGCCTTGACC-3’; 5’-TTGGAGTGGTCAGCGTAAGT-3’; *M*-cKO-Exon3-4: 5’-GCTGTGGCAGAAAAGAAAGGTC-3’; 5’-CTGTTCCTTGGCTGTTGTGGTA-3’; *F*-cKO-Exon1-2: 5’-GACACTTGGCTTCCTTACCTG-3’; 5’-CTCACCACGTCCCGAAACAA-3’; *F*-cKO-Exon2-3: 5’-TTCATGCTGGATGACCTCAATG-3’; 5’-GCCAACTGACAGCGTTCTAAG-3’; U6: 5’-ATGGGTCGAAGTCGTAGCC-3’; 5’-TTCTCGGCGTCTTCTTTCTCG-3’.

### BrdU labeling assay

Time-pregnant females were injected intraperitoneally with a single pulse BrdU (Sigma-Aldrich, St Louis, MO, USA) (50 μg/g body weight) at E13.5 and E15.5. Brain tissues were collected after 1 h and followed with BrdU antibody staining.

### Tissue preparation and immunohistochemistry

Mouse brains were fixed in 4% PFA in phosphate-buffered saline (PBS) overnight, incubated in 25–30% sucrose in PBS, embedded in OCT (SAKURA, USA), and stored at –80 °C until use. Brains were sectioned (14–16 μm) using a cryostat. For antigen recovery, sections were incubated in heated (95–100 °C) antigen recovery solution (1 mM EDTA, 5 mM Tris, pH 8.0) for 15–20 min, and cooled down for 20–30 min. Before applying antibodies, sections were blocked in 10% normal goat serum in PBS with 0.1% Tween-20 (PBT) for 1 h. Sections were incubated with primary antibodies at 4 °C overnight and visualized using goat anti-rabbit IgG–Alexa-Fluor-488 (Jackson ImmunoResearch, UK) and/or goat anti-mouse IgG–Alexa-Fluor-546 (Jackson ImmunoResearch, UK) (1:300, Molecular Probes) for 1.5 h at room temperature. Images were captured using a Leica digital camera under a fluorescent microscope (Leica DMI6000B).

Primary antibodies against the following antigens were used: BrdU (1:100) (Abcam, UK), Ki67 (1:300) (Abcam, UK), Sox2 (1:100) (Santa Cruz Biotechnology, USA), Pax6 (PRB-278P, 1:200, BioLegend), Tbr1 (1:2,000) (Abcam, UK), Tbr2 (1:500) (Abcam, UK), NeuN (1:3,000) (Abcam, UK), Satb2 (1:500) (Abcam, UK), Caspase3 (1:100, Abcam), and DAPI (D9542, 1:1000, Sigma), also see Supplementary Table S14.

### Nissl staining

Brain sections (14 μm) were processed through incubation in the following solutions in this order: 100% ethanol (30 s), 95% ethanol (30 s), distilled water (30 s, twice), cresyl violet (Sigma-Aldrich, St Louis, MO, USA) (3–5 min), distilled water (2 min, three times), 50% ethanol (2 min), 95% ethanol (5–30 min), 100% ethanol (5 min, twice), and xylene (MACKLIN, Shanghai, China) (3 min, twice). Thereafter, the sections were mounted with a coverslip.

### Cell counting in the cortical wall

Coronal brain sections of medial cortical regions (between the anterior commissure and the anterior hippocampus) were collected. At least four sections from each brain and three different mouse litters were selected for antibody labeling. Positive cells were quantified in fixed areas of 75, 350, 200, and 300 mm bins for E13.5, E15.5, P0, and P14 cortices, respectively. The statistical results were plotted using Graphpad Prism 7.

### RNA sequencing (RNA-seq)

One microgram total RNA per sample was used for sequencing library preparation using the NEBNext^®^ UltraTM RNA Library Prep Kit from Illumina^®^ (NEB, USA) following manufacturer’s instructions, and index codes were added to attribute sequences to each sample. PCR products were purified (AMPure XP system) and library quality was assessed on the Agilent Bioanalyzer 2100 system. The clustering of the index-coded samples was performed on a cBot Cluster Generation System using TruSeq PE Cluster Kit v3-cBot-HS (Illumina, USA). The library preparations were sequenced on an Illumina Novaseq platform and 150 bp paired-end reads were generated.

Raw data (raw reads) of fastq format were processed through in-house perl scripts. Clean data (clean reads) were obtained by removing reads containing adapter, reads containing ploy-N and low quality reads. All downstream analyses were based on the clean data. Paired-end clean reads were aligned to the reference genome using Hisat2 v2.0.5. Raw data were deposited in Gene Expression Omnibus (GEO) under the series number GSE154992. Index of the reference genome was built using Hisat2 v2.0.5.

### Ribosome sequencing (Ribo-seq)

Three E15.5 mouse cerebral cortical samples from 1 L were treated with lysis buffer with cycloheximide (50 mg/mL) to acquire the lysate. The tissue lysate was treated with unspecific endoribonuclease RNase I (Ambion, USA). Following PAGE purification, both ends of RPFs were phosphorylated and ligated with 5’ and 3’ adapters. The fragments were then reversely transcribed to cDNAs, amplified by PCR, and subjected for sequencing.

TopHat2 was used for genome mapping in Ribo-seq. Quantification of mapped results to the gene level was carried out using HTSeq. “Reads Per Kilobase of transcript per Million mapped reads” (RPKM) values were generated to represent the gene expression level of each specific gene.

TE was calculated as the ratio of RPKM in Ribo-seq to RPKM in RNA-seq. Differential TE analysis was performed using RiboDiff for samples with biological replicates. The expression distribution map was drawn using ggplot2 R package.

### Quantification of integrative genomics viewer (IGV)

In RNA-seq and Ribo-Seq, RPKM values of the triad sequencing results in each group were collected from three samples. The average RPKM value of three samples in the control group was used as the common denominator, and average RPKM values in the *M*-cKO, *F*-cKO, and control were used as the numerator for normalization. All results are presented as mean ± standard error of the mean (SEM).

### Statistical analysis

All experiments using mouse brains were repeated at least with three biological replicates. Non-normally distributed continuous data were presented as medians with interquartile range and compared using the Mann–Whitney *U* test. The survival data were analyzed by the Kaplan–Meier test. All results were presented as mean ± SEM *P* values were determined by the unpaired Student’s *t*-test, and *P* values < 0.05 were considered significant. The resulting *P* values were adjusted using Benjamini and Hochberg’s approach for controlling the false discovery rate. Genes with |log2 (Fold Change)| > 0 and *P*_adj_ < 0.05 were assigned as differentially expressed.

## Supplementary information

Supplementary Figures

Supplementary Table S1

Supplementary Table S2

Supplementary Table S3

Supplementary Table S4

Supplementary Table S5

Supplementary Table S6

Supplementary Table S7

Supplementary Table S8

Supplementary Table S9

Supplementary Table S10

Supplementary Table S11

Supplementary Table S12

Supplementary Table S13

Supplementary Table S14

## Data Availability

All high-throughput sequencing data generated in this study (RNA-seq and Ribo-seq) have been deposited in GEO (Accession number GEO: GSE154992).

## Code availability

No custom code was generated in this study.
