# Supplementary material for: Distinct roles of Fto and Mettl3 in controlling development of the cerebral cortex through transcriptional and translational regulations
Source: Cell Death Dis. 2021 Jul 14;12(7):700. doi: 10.1038/s41419-021-03992-2 (PMC8280107; doi:10.1038/s41419-021-03992-2)
Supplement: Supplementary file 1 — Supplementary Figures [file 41419_2021_3992_MOESM1_ESM.pdf]

Supplementary data

Distinct roles of Fto and Mettl3 in controlling development of the cerebral cortex through transcriptional and translational regulations

Kunzhao Du[1,#], Zhen Zhang[2,#], Zhiwei Zeng[1], Jinling Tang[1], Trevor Lee[3] and Tao Sun[1,*]

[1]Center for Precision Medicine, School of Medicine and School of Biomedical Sciences, Huaqiao University, Xiamen, Fujian 361021, China

[2]School of Life Sciences and Biotechnology, Shanghai Jiao Tong University, Shanghai 200240, China

[3]Department of Cell and Developmental Biology, Cornell University Weill Medical College, 1300 York Avenue, New York, NY 10065, USA

[*]Corresponding author: Dr. Tao Sun, Email: taosun@hqu.edu.cn.

[#]These authors contributed equally to this work.

Running title: Mettl3 and Fto control cortical development.

Supplementary Figures

Figure S1

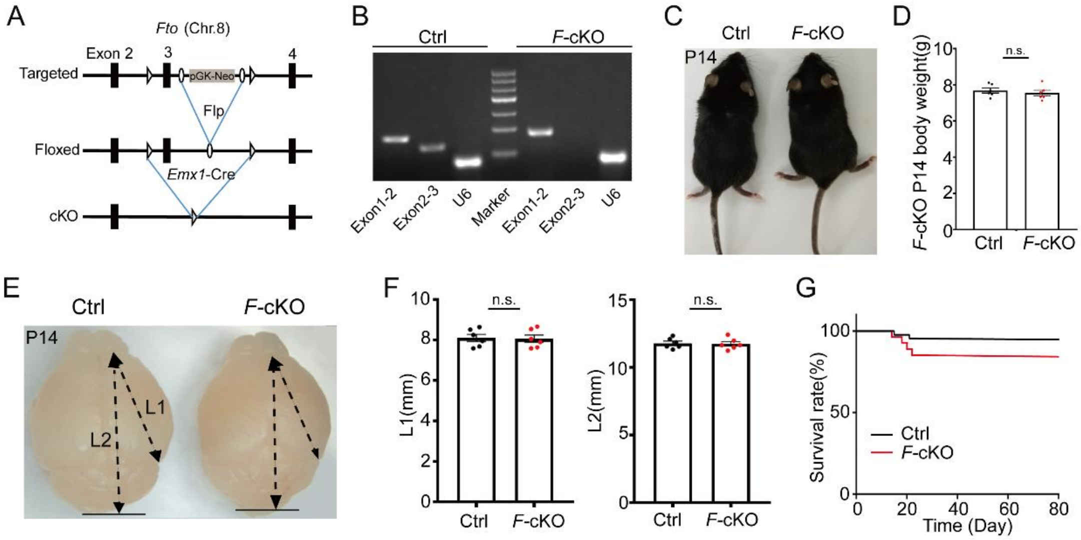

Figure S1. Cortical specific knockout of *Fto* doesn't cause detectable brain defects. (**A**) Strategy of generating cortical specific *Fto* knockout mice, named *F*-cKO, depletion of *Fto* gene in the cerebral cortex by breeding floxed *Fto* mice with *Emx1-Cre* mice. (**B**) The knockout efficiency was detected by reverse transcription PCR using RNA from E13.5 control (Ctrl) and *F*-cKO mouse cerebral cortices. (**C** and **D**) Body weight of *F*-cKO mice at P14 was not changed compared to the Ctrl. (**E** and **F**) The brain size was not changed in *F*-cKO mice at P14. "L1" represents the cortical length, and "L2" represents the brain length. (**G**) Survival curve of Ctrl (n = 48) and *F*-cKO (n = 24) pups. Scale bar: 1mm. Error bars indicate the s.e.m. (six independent samples). *P*-values were calculated by Student's t-test between Ctrl and *F*-cKO. *P*-values: n.s.: non-significant.

Figure S2

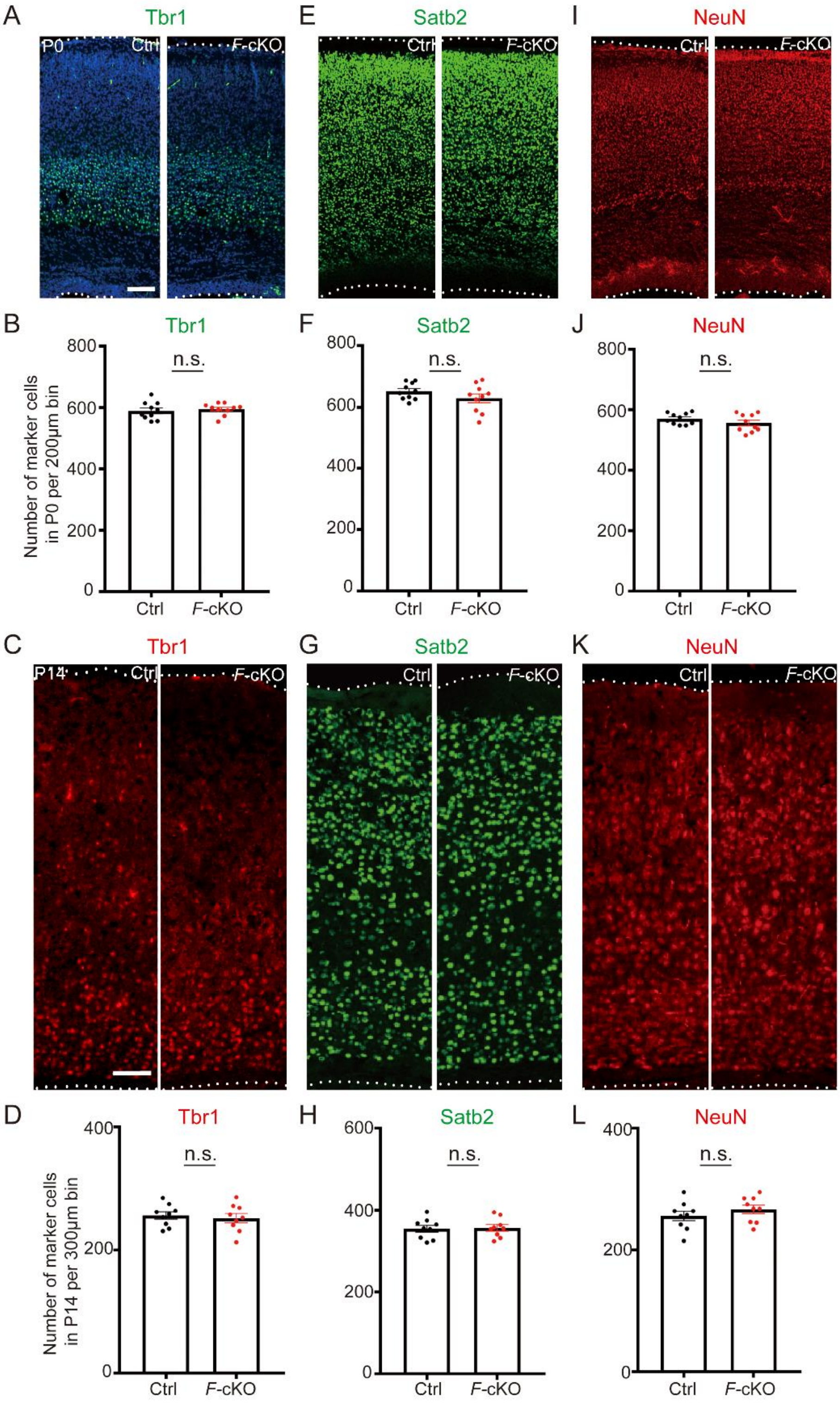

Figure S2. Deletion of *Fto* in the cerebral cortex does not alter neuronal production. (**A-D**) The numbers of Tbr1$^+$ neurons were not changed in P0 (A, B) and P14 (C, D) *F*-

cKO cortices, compared to controls (Ctrl). (**E-H**) The numbers of Satb2$^+$ neurons were not changed in P0 (E, F) and P14 (G, H) *F*-cKO cortices, compared to the Ctrl. (**I-L**) The numbers of NeuN$^+$ neurons were not changed in P0 (I, J) and P14 (K, L) *F*-cKO cortices, compared to the Ctrl. Scale bars: 100μm. Error bars indicate the s.e.m. (five independent samples). *P*-values were calculated by Student's t-test between Ctrl and *F*-cKO. *P*-values: n.s.: non-significant.

Figure S3

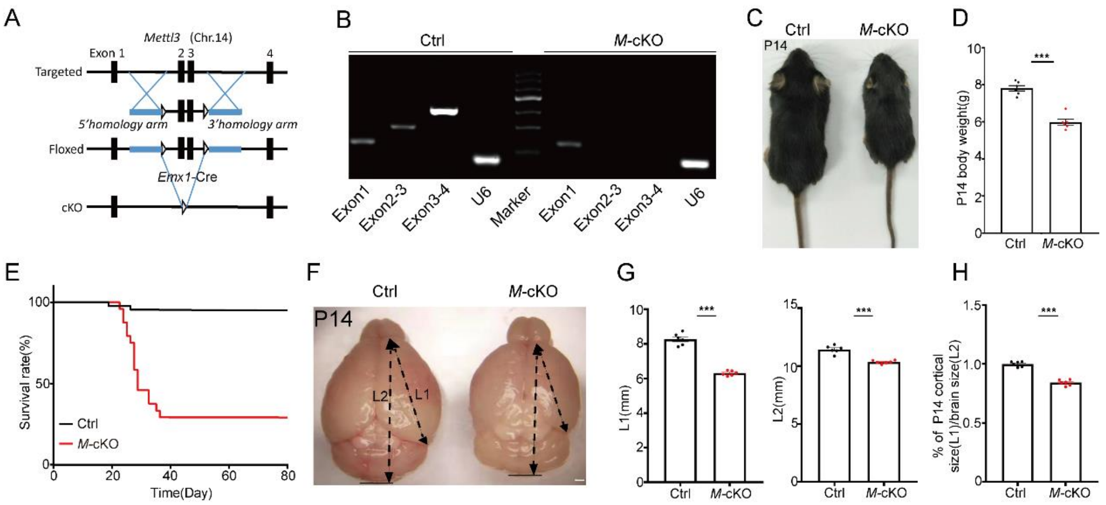

Figure S3. Cortical specific knockout of *Mettl3* results in significant reduction of body and brain size. (**A**) Strategy of generating cortical specific *Mettl3* knockout mice, named *M*-cKO, depletion of *Mettl3* gene in the cerebral cortex by breeding floxed *Mettl3* mice with *Emx1-Cre* mice. (**B**) The knockout efficiency was detected by reverse transcription PCR using RNA from E13.5 control (Ctrl) and *M*-cKO mouse cerebral cortices. (**C** and **D**) Body weight of *M*-cKO mice at P14 was significantly reduced compared to the Ctrl. (**E**) Survival curve of Ctrl (n = 48) and *M*-cKO (n = 23) pups. (**F** and **G**) The brain size was reduced in *M*-cKO mice at P14. "L1" represents the cortical length, and "L2" represents the brain length. (**H**) The percentage of P14 cortical size (L1)/Brain size (L2). "L1" represents the cortical length, and "L2" represents the brain length. Scale bars: 1mm. Error bars indicate the s.e.m. (six independent samples). *P*-values were calculated by Student's t-test between Ctrl and *M*-cKO. *P*-values: *** $P < 0.001$.

Figure S4

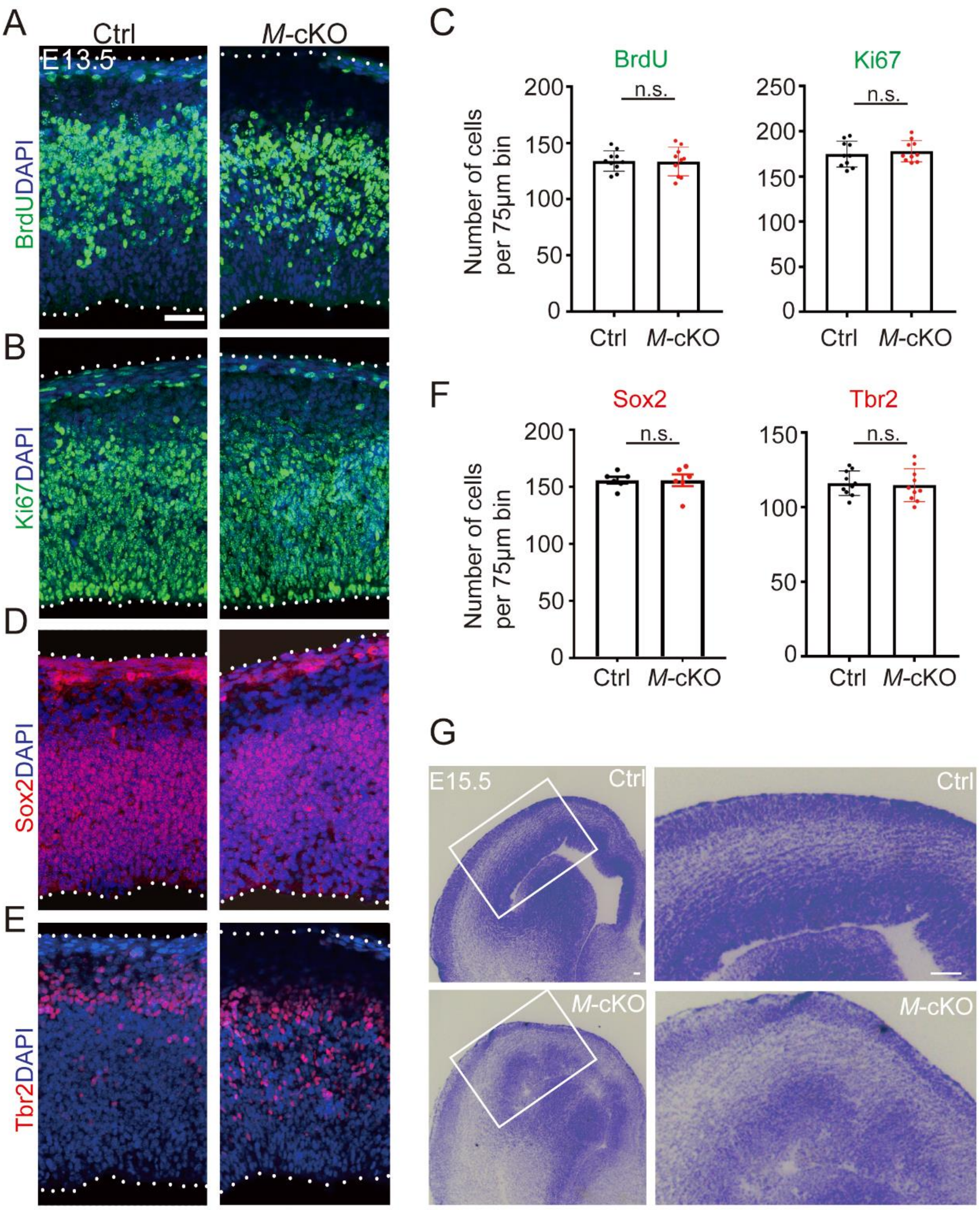

Figure S4. Knockout of *Mettl3* in E13.5 and E15.5 cerebral cortices. (**A-C**) The numbers of BrdU^{+} and Ki67^{+} cells were not changed in *M*-cKO cortices, compared to controls (Ctrl). (**D-F**) The numbers of Sox2^{+} and Tbr2^{+} neural progenitors were not changed in *M*-cKO cortices, compared to the Ctrl. (**G**) Nissl staining of E15.5 Ctrl and *M*-cKO mouse brain sections. Regions in white boxes are shown at a higher

magnification (right). Scale bars: 100μm. Error bars indicate the s.e.m. (five independent samples). *P*-values were calculated by Student's t-test between Ctrl and *M*-cKO. *P*-values: n.s.: non-significant.

Figure S5

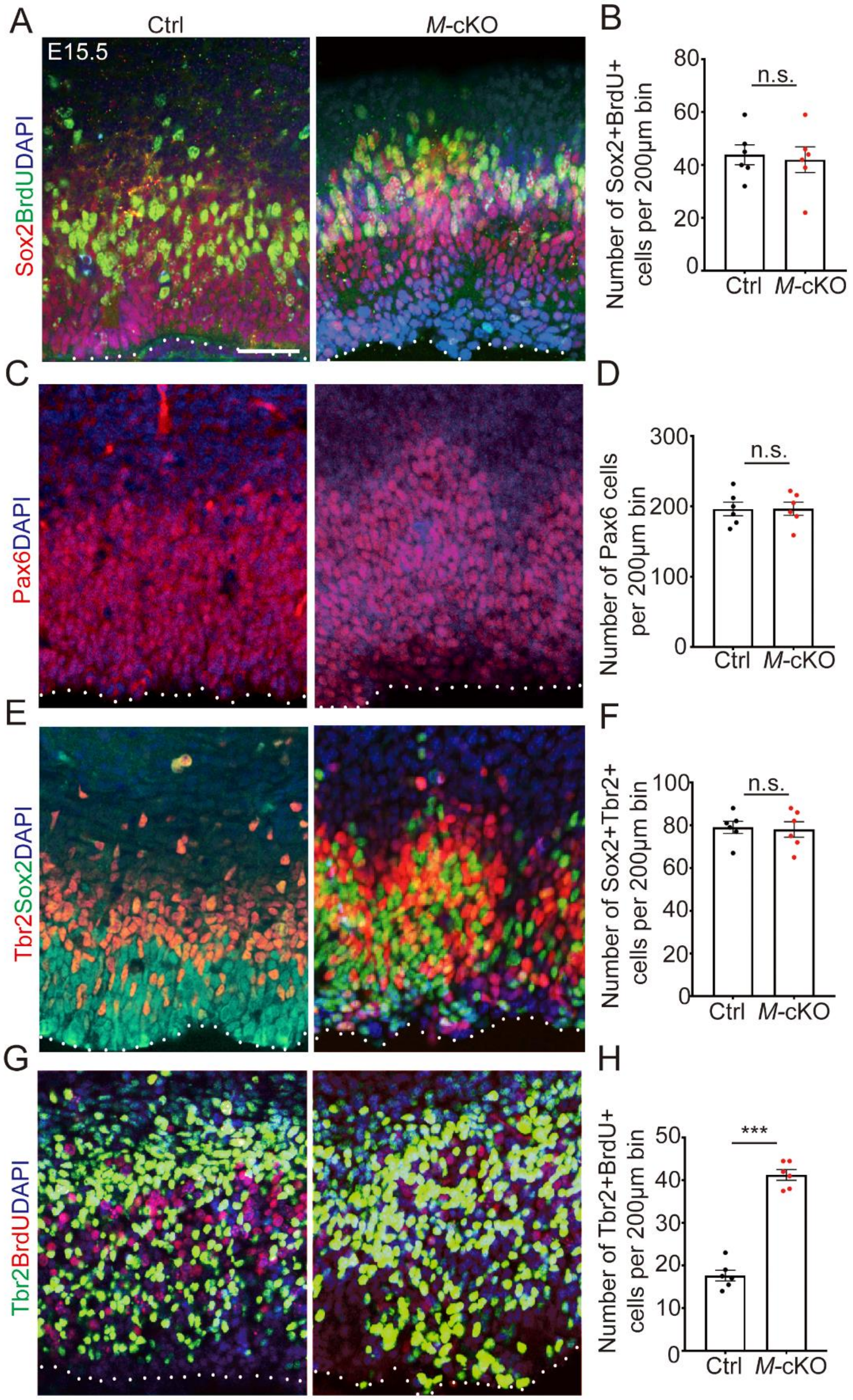

Figure S5. The deletion of *Mettl3* resulted in expanded intermediate progenitors (IPs) in the cerebral cortex of E15.5 mice. (**A-B**) The numbers of $Sox2^{+}BrdU^{+}$ proliferating

RGCs were not changed in *M*-cKO cortices. (**C-D**) The numbers of $Pax6^+$ RGCs were not changed in *M*-cKO cortices. (**E-F**) The numbers of $Sox2^+Tbr2^+$ cells, reflecting a transition from radial glial cells (RGCs) to IPs, were not changed in *M*-cKO cortices. (**G-H**) The numbers of $Tbr2^+BrdU^+$ proliferating IPs were increased in *M*-cKO cortices, compared to the controls (Ctrl). Scale bar: 50μm. Error bars indicate the s.e.m. (six independent samples). *P*-values were calculated by Student's t-test between Ctrl and *M*-cKO. *P*-values: n.s.: non-significant; *** $P < 0.001$.

Figure S6

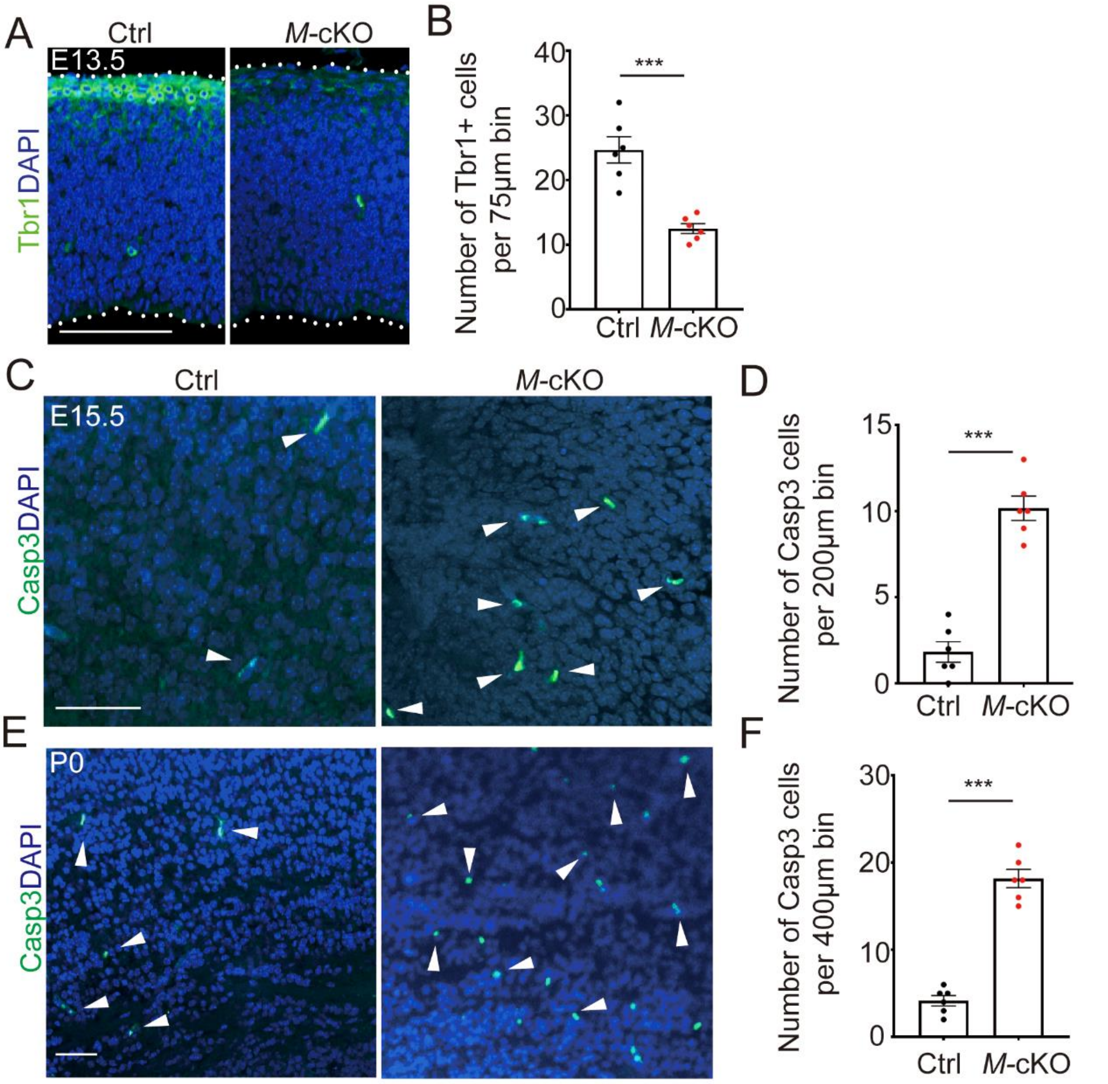

Figure S6. The deletion of Mettl3 promotes cell apoptosis in the cerebral cortex. (**A-B**) The numbers of Tbr1$^+$ deep layer neurons were reduced in E13.5 *M*-cKO cortices, compared to the controls (Ctrl). (**C-F**) The numbers of Casp3$^+$ apoptotic cells were increased in E15.5 (**C** and **D**) and P0 (**E** and **F**), compared to the Ctrl. Arrowheads indicate apoptotic cells. Scale bars: 50μm. Error bars indicate the s.e.m. (six independent samples). *P*-values were calculated by Student's t-test between Ctrl and *M*-cKO. *P*-values: *** $P < 0.001$.

Figure S7

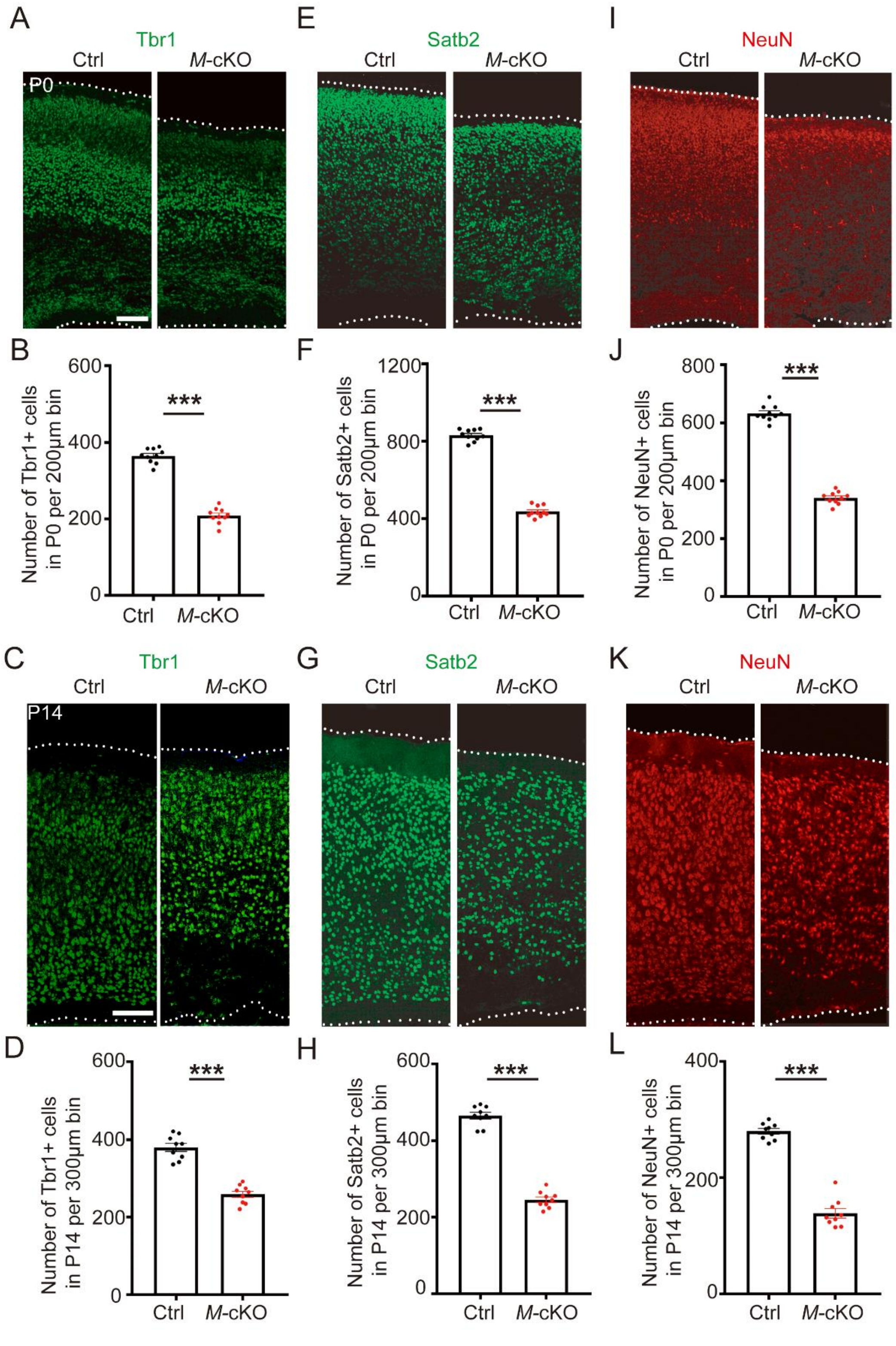

Figure S7. Cortical deletion of *Mettl3* results in decreased neuronal production. (**A-D**) The numbers of $Tbr1^{+}$ neurons were reduced in P0 (A, B) and P14 (C, D) *M*-cKO

cortices, compared to the control (Ctrl). (**E-H**) The numbers of Satb2^{+} neurons were reduced in P0 (E, F) and P14 (G, H) *M*-cKO cortices, compared to the control. (**I-L**) The numbers of NeuN^{+} neurons were reduced in P0 (I, J) and P14 (K, L) *M*-cKO cortices, compared to the Ctrl. Scale bars: 100μm. Error bars indicate the s.e.m. (six independent samples). *P*-values were calculated by Student's t-test between Ctrl and *M*-cKO. *P*-values: *** $P < 0.001$.

Figure S8

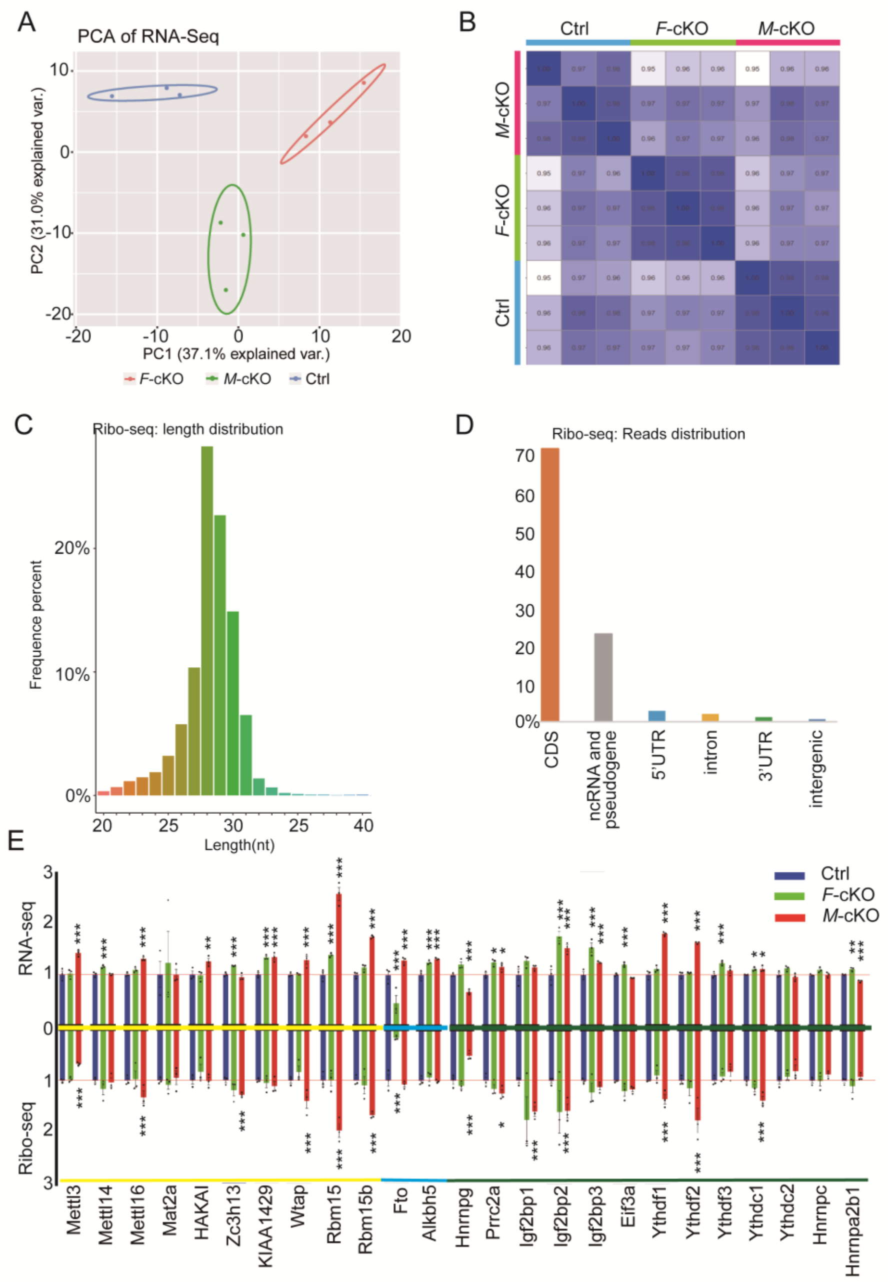

Figure S8. Sequencing quality control of RNA-seq and Ribo-seq. (**A** and **B**) Both principal component analysis (PCA) (A) and Pearson correlation coefficient (B) showed good repeatability in three replicate samples in RNA-seq. (**C**) Volcano map of up- and down-regulated genes in *F*-cKO cortices, compared to the control (Ctrl), detected by RNA-seq. (**D**) Sequencing length distribution and RNA transcript locations detected by Ribo-seq. (**E**) Expression quantifications of major components in m^{6}A

Methylation in E15.5 control (Ctrl), *F*-cKO and *M*-cKO cortices detected by RNA-seq and Ribo-seq. “Writers” (yellow), “erasers” (blue) and “readers” (green) are illustrated. Error bars indicate the s.e.m. (three independent samples). *P*-values were calculated by Student’s t-test between Ctrl and *F*-cKO or Ctrl and *M*-cKO. *P*-values: * $P < 0.05$; ** $P < 0.01$; *** $P < 0.001$.

Figure S9

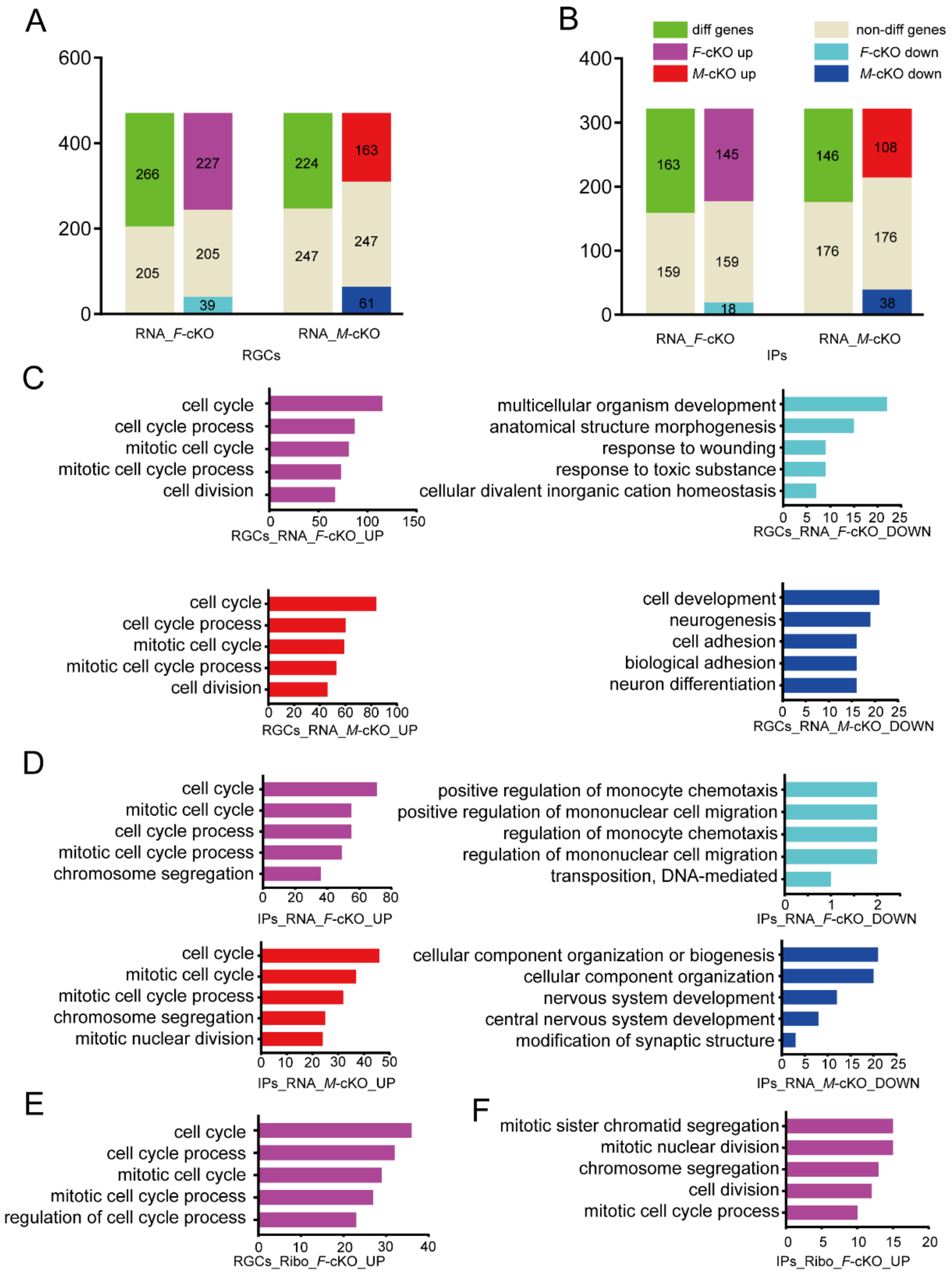

Figure S9. Different expressions of RGCs and IPs gene pools in *Mettl3* and *Fto* deletion. (**A** and **B**) RGCs (A) and IPs (B) gene pool expression at transcriptome sequencing after *Mettl3* and *Fto* deletion. Diff genes: the number of genes that are significantly different, non-diff genes: the number of genes with no significant difference, *F*-cKO up

and *M*-cKO up: the number of genes that are up-regulated in *F*-cKO and *M*-cKO, *F*-cKO down and *M*-cKO down: the number of genes that are down-regulated in *F*-cKO and *M*-cKO. (**C** and **D**) GO analyses showed top biological process enriched terms of RGCs (C) and IPs (D) under different regulatory conditions in RNA-seq. The color of (C) corresponds to that of (A), (D) corresponds to (B). (**E** and **F**) GO analyses showed top biological process enriched terms in RGCs (E) and IPs (F) in up-regulated genes in *F*-cKO cortices detected by Ribo-seq.
